# Inter-Individual Variability and Conspecific Densities: Consequences for Population Regulation and Range Expansion

**DOI:** 10.1371/journal.pone.0033375

**Published:** 2012-03-27

**Authors:** Laura Cardador, Martina Carrete, Santi Mañosa

**Affiliations:** 1 Departament de Biologia Animal, Universitat de Barcelona, Barcelona, Spain; 2 Department of Physical, Chemical and Natural Systems, Universidad Pablo de Olavide, Sevilla, Spain; Phillip Island Nature Parks, Australia

## Abstract

The presence of conspecifics can strongly modulate the quality of a breeding site. Both positive and negative effects of conspecifics can act on the same individuals, with the final balance between its costs and benefits depending on individual characteristics. A particular case of inter-individual variation found in many avian species is chromatic variability. Among birds, plumage coloration can co-vary with morphology, physiology and behavior as well as with age. These relationships suggest that cost-benefit balances of conspecific presence may be different for individuals with different colorations. We investigated whether inter-individual variability affects population regulation and expansion processes by analyzing potential differences in density-dependent productivity and settlement patterns in relation to plumage coloration in a population of a long-lived avian species recently undergoing a notable increase in numbers and distribution range. Our results show strong variation in the effect of density on productivity of breeding pairs depending on plumage coloration of their members. Productivity of dark birds decreased along the breeding density gradient while that of lighter breeders remained unchanged with conspecific density. In a similar way, our results showed an uneven occupation of localities by individuals with different plumage coloration in relation to local densities, with the breeding of lighter harriers more aggregated than that of dark-brown ones. At a population scale, darker birds had higher probability of colonization of the most isolated, empty sites. Explanations for species range expansion and population regulation usually make the inferred assumption that species traits are similar among individuals. However, in most species, there could be individual variation in niche requirements or dispersal propensities among individuals with different traits. Our results contribute to the growing appreciation that the individual traits, but not the average trait at the level of species, are important during population regulation and expansion processes.

## Introduction

Decisions taken by animals when selecting a particular site at which to settle have important consequences for their fitness. Both empirical and experimental studies have shown the effect of site quality, in terms of habitat characteristics, resource availability or presence of predators and/or parasites, on occupancy patterns and fitness components [1]–[4]. In particular, the presence of conspecifics can strongly modulate the quality of a breeding site [5]–[7]. Given that conspecifics are competitors, the addition of every new individual to a patch may decrease its suitability [8] or even prevent its occupation by other individuals [4], [9], [10]. The remaining capacity of a site to support further settlement would thus result from an interaction between habitat characteristics as well as the presence and number of conspecifics. In this case, when population size increases, average fitness within the population is expected to decrease as a consequence of a monotonic impoverishment of all breeding sites (Ideal Free Distribution) [8] or because an increasing proportion of the population is displaced into poorer-quality sites (Ideal Despotic and Site-dependent Distributions) [8], [9]. However, conspecifics can also provide advantages for settlers such as transmission of information about food, earlier predator detection, higher defense efficiency or more mating opportunities [5], [11], [12], which should have benefits for individual fitness [13], [14]. Both positive and negative effects of conspecifics are usually present in the same population and even act on the same individuals [15], thus leading to a trade-off between conspecifics' cost and benefits [5], [12], [16]. If positive and negative effects of density at the breeding sites exist, individuals may move away from both low and high density areas [17], with settlement patterns depending on both individual characteristics and external conditions [15], [18].

Although site quality is a major determinant of fitness, its effect can be confounded by individual quality, a relationship that has been little studied in large, long-lived vertebrates [19]. Individual differences in behavior, morphology, physiology and life history traits are common across taxa and may have consequences for population dynamics and regulation. For example, empirical evidence suggests that density-dependent effects on individual fitness differed among individuals in relation to their sex or age [20], [21]. Another case of inter-individual variation found in many species is chromatic variability. Among birds, color polymorphism is known to co-vary with morphology, physiology and behavior [22]–[24]. In particular, melanin-based coloration has been associated with aggressiveness [25], [26], a relationship that results from pleiotropic effects of the genes regulating the synthesis of this pigment [23]. Color variability might thus modulate intraspecific interactions [27], leading to differential cost-benefit balances of conspecific presence for individuals with different colorations. Though several studies have focused on color polymorphism and its associated function [e.g. 22]–[24], [ 28], little is known about density-dependent effects on fitness changes among individuals showing different plumage color [29].

Here, we investigated whether color variability between individuals has consequences on fitness and on population regulation processes in a long-lived avian species. We used as a study model the marsh harrier *Circus aeruginosus*, a ground-nesting raptor that can breed solitarily or by forming sparse colonies in natural or artificial wetlands [30], [31]. This species presents strong sexual dichromatism as well as different plumage coloration, which is presumably melanin-based [26]. Two male colorations have been described, with males being either ‘grey’ or ‘brown’ [26], [32], [33]. Moreover, individuals show variation in general coloration, between dark and light, but also becoming lighter with age [26], [34]. Focusing on productivity, we predict that because of birds have different competitive skills in relation to their coloration (i.e., grey individuals are more aggressive than brown individuals) [26], the result of conspecific density on productivity would vary among breeding pairs depending on the plumage coloration of their members. In this case, we could expect that breeders of different colorations do not distribute equally among localities with different levels of conspecific density, so less competitive individuals would avoid settlement in high density areas to maximize their fitness. This would occurs because in heterogeneous environments settlement behavior should be selected to increase individual fitness by favoring the displacement of animals to suitable patches [16], [29]. If this pattern of avoidance of high local conspecific densities shown by some individuals translates to a larger scale, we can thus expect that the probability of colonization of new isolated breeding sites at the population scale varied in relation to plumage coloration. It should be noted that since coloration in marsh harriers is also linked to age, younger individuals being dark while adults tend to be lighter [34], it is difficult to completely disentangle polymorphism from age. However, whatever the cause of these individual differences (polymorphisms or age), trends between productivity and conspecific densities should be similar, younger/darker individuals being less competitive than adult/light ones and thus searching for less aggregated nest-sites to increase their breeding output [15].

## Methods

### Ethics Statement

Censuses were performed within the monitoring program for the marsh harrier of the Departament de Medi Ambient de la Generalitat de Catalunya (autonomic government, permits SF/182, SF/118, SF/173). The field work procedures were not invasive and did not involve entering into any protected area or area restricted to public use and did not require additional permits by the relevant Spanish authorities.

### Study area and species

**Figure 1:**
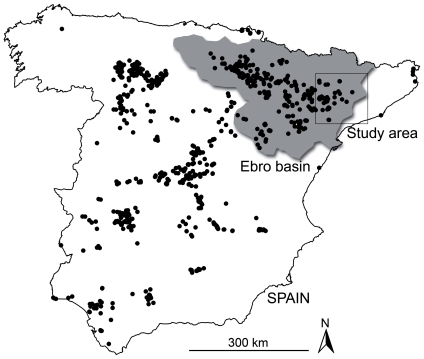
Study area and distribution of the breeding population of the marsh harrier in Spain in 2006 [**39**]. The Ebro Basin is shown in grey.

Field work was carried out in a large agricultural area in the Catalan Ebro Basin, in north-eastern Spain (Lleida region, 7300 km^2^, Fig. 1). This is an agricultural mosaic of field crops and natural and artificial wetlands (ponds and reservoirs related to agricultural practices) composed of a mixture of arable, non-irrigated cereal fields (mainly wheat and barley), irrigated fields (mainly alfalfa, wheat, barley and some sweet corn), dry fruit trees (mainly olive and almond trees) and irrigated fruit trees (mainly peach, pear, apple and nectarine trees). The landscape is mostly low-lying and flat, broken by discrete ranges of small hills (0–400 m a.s.l.) with a semiarid Mediterranean climate.

The marsh harrier *Circus aeruginosus* is a medium-sized, semi-colonial raptor that mainly breeds in wetlands, behaving as an open-habitat hunter [30]. Previous works in the study area show that they mainly use wetlands and their associated emergent vegetation for breeding, while the surrounding crops constituted their foraging areas [31], [35], [36]. The European marsh harrier population underwent a dramatic population decline from 1960 to 1980, but recovered in recent decades when the species experienced around 10% increase [30], [37]. This tendency was also observed in the study area, where the species has increased both in numbers (from 8 to 51 breeding pairs) and distribution range (from ca. 60 to 3,000 km^2^) from 1997 to 2008 [31].

### Census and reproductive data

The breeding population of marsh harriers in the study area has been monitored since 1997, with plumage color and productivity of breeding pairs accurately assessed for the period 2001–2008. Each year, occupancy was determined by repeated visits to known breeding sites as well as to other potentially suitable areas, looking for signs of breeding behavior such as territorial displays or deliveries of nest material or prey items to the nest. Potentially suitable breeding sites were considered to include all wetlands (defined as artificial ponds, reservoirs, marshes and parts of rivers or watercourses) [31] with the presence of tall standing reeds *Phragmites australis*, reedmace *Typha angustifolia* or other dense emergent aquatic vegetation (the nesting habitat of the species) [30]. In total, 51 breeding sites were visited. Observations were made from vantage points located >100 m from the nests by using 10×42 binoculars and 20–60× telescopes. Due to the difficulty in identifying individuals of each pair before females began laying, especially at breeding sites where more than one pair breeds, we considered as breeding pairs only those in which we confirmed that females had begun laying eggs. Visits began in early April, when nest building started, and continued until incubation (April–May). Successive visit intervals were around 1.5 weeks. For each breeding pair we derived mean laying date as the mean date between the first visit when incubation was confirmed and the previous one. We also determined the incidence of polygamous relationships among all parents in breeding locations by long continuous observations of male parents bringing material to the nests and food to the females and nestlings. Individual identification - necessary to establish polygamy - was possible due to the considerable variation in male plumage and color pattern [38]. A location was considered to be unoccupied if no individuals with signs of breeding behavior were observed in three visits of 1.5 hours to potential breeding sites between April–May. Visits were conducted only in periods with good weather conditions (with no rain or wind). They were conducted between sunrise and sunset, avoiding hours of maximum heat (13:00–16:00 approximately). During the first days of fledging, approximately 70 days after incubation, occupied breeding sites were visited again to record the number of fledglings per nest (mid June–August).

### Bird coloration

General coloration of breeders was assessed during censuses of breeding pairs. Males were easily classified into three color categories, ‘dark brown’, ‘light brown’ and ‘grey’ according to the presence or absence of grey feathers in the upper-wing and on whether the under-wing appeared completely dark brown or light [26], [34]. Females were classified as ‘dark’ or ‘light’ according to whether the upper-wing was completely dark brown or light brown with very large and apparent white shoulders [26], [34]. Although females and brown males look alike, they were easily recognized by their different roles in reproduction (i.e., only females incubate) [30]. Differences in coloration in relation to age may hinder the differentiation of ‘brown’ adults from subadults in both males and females. Previous information shows that age of first breeding in this species is 2–3 years (for females and males, respectively) [39], while younger individuals (<2 or <3 years for females and males, respectively) entering into the breeding population is extremely rare [30], [39]. However, as our population is increasing, we cannot discard dark individuals as being young breeders [40].

### Breeding densities

Breeding densities were annually measured for each pair through an aggregation index describing its relative position within the spatial distribution of the breeding population (population aggregation) and within the spatial distribution of pairs occupying the same locality (local aggregation). Aggregation indexes were calculated as *∑exp(−d_ij_)* (with *i≠j*), where *d_ij_* was the linear distance between breeding pair *i* and *j*, *j* representing all breeding pairs within a locality (local aggregation) or all known breeding pairs (population aggregation) [21]. Because our marsh harrier breeding population was the continuation of a larger breeding population located in the west of the Ebro Basin (Fig. 1), for calculations of annual population aggregation we included information on the spatial distribution of this western population. Although accurate information of the marsh harrier breeding population in the surroundings of the study area was only available for 2006 (data from national census of SEO/BirdLife), the global geographic distribution of the population has remained unchanged during recent years (based on the comparison between national censuses from 1998 and 2006) [41], [42], so we used the same data for the western population for all years [31]. In this way, population aggregation values took into account most of the relevant marsh harrier breeding locations both within and outside of our study area. However, local and population aggregation indexes resulted highly correlated (Pearson correlation almost 1), showing that population aggregation indexes were strongly influenced by the distribution of the closest breeding pairs.

### Statistical analysis

#### Bird coloration

We tested for potential differences in plumage color scores assigned by different observers to the same birds by using a collection of 20 harrier's photographs representative of plumage coloration of the species. We used generalized linear mixed models with a multinomial error distribution and a cumlogit link function for the dependent ordinal variable (plumage score). We fitted observer as a fixed effect and harrier and sex as random terms in the model.

#### Bird coloration, local conspecific density and breeding performance

We employed generalized linear mixed models (GLMMs) with a Poisson error distribution and a log link function to analyze factors affecting productivity (i.e. number of young fledged per breeding pair per year). Some studies suggest that individual and habitat quality could be coupled, high quality individuals breeding in high quality areas, so that habitat heterogeneity but not inter-individual variability may explain productivity differences [2], [43]. Thus, we first assessed whether productivity of breeding pairs in our study area was affected by potential differences among localities by running a GLMM with the single effect of ‘locality’ as a fixed term. Afterwards, we run another GLMM to evaluate whether productivity of breeding pairs varied according to male and female plumage coloration (fitted as ordinal variables, with male plumage categories ‘grey’>‘light brown’>‘dark brown’; and female categories ‘light’>‘dark’) or to a combination of both (interaction ‘male coloration×female coloration’). Finally, we analyzed whether productivity of breeding pairs was affected by conspecific aggregation by fitting the single effect of local conspecific aggregation as an independent variable (in both its linear and quadratic forms) in another GLMM. After that, we combined all potential effects (i.e. locality, male and female plumage colorations and conspecific aggregation) as well as the interaction between male and female plumage coloration and conspecific aggregation in the same GLMM to assessed the relative contribution of these variables on reproduction and whether productivity of breeding pairs composed of individuals with different coloration were differently affected by local conspecific aggregation. We control for potential differences in productivity among breeding pairs and trios [38] by including as a fixed effect a categorical variable with two levels (polygynous vs. non-polygynous male) in all models [21]. “Year” was also included as a random effect in all models to control for non-independence of data.

#### Bird coloration, settlement pattern and population expansion

We looked for differences in settlement patterns among breeding pairs composed of individuals with different plumage coloration at a local scale and whether it translates into differential probabilities of colonization of the most isolated empty sites at the population scale. For this purpose, we first analyzed whether local aggregation varied among breeding pairs in relation to their coloration by fitting male and female plumage coloration and their interaction as fixed effects and local aggregation as the dependent variable in a GLMM. Since this local aggregation index highly deviated from normality, we transformed it into a categorical ordered variable with ten levels (by rounding off aggregation values) to use a multinomial error distribution and a cumulative logit link function for model construction. We controlled for polygyny as a fixed effect and year as a random term in this model (see above). After that, we investigated whether the probability that a breeding pair colonized a new location (i.e., settled at a previously empty location vs. settled at a location that had been used in the previous year) at a population scale varied in relation to male and female plumage coloration, habitat characteristics (i.e. locality), population aggregation at that site or to a combination of plumage coloration and population aggregation. We did so by constructing a GLMM with occupation of empty sites as a response variable (binomial error distribution and logit link function) and plumage coloration of male and females, locality, population aggregation and the potential interaction between male and female plumage coloration and aggregation as independent variables. We controlled for polygyny as a fixed effect and year as a random term in this model as explained before. All analyses were performed by using SAS 9.2 (SAS Institute Inc., 2009).

## Results

**Figure 2:**
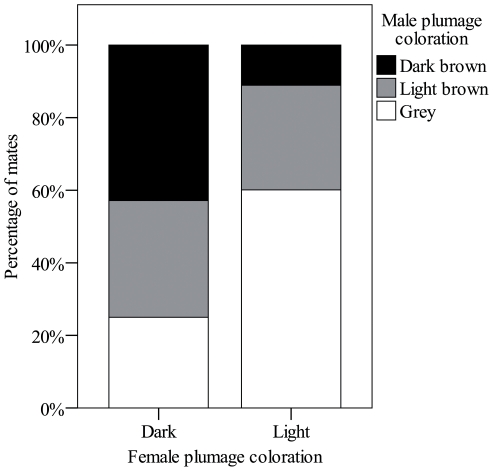
Relationship between male and female plumage coloration of marsh harrier breeding pairs (Ebro basin, NE Spain, 2001–2008).

**Figure 3:**
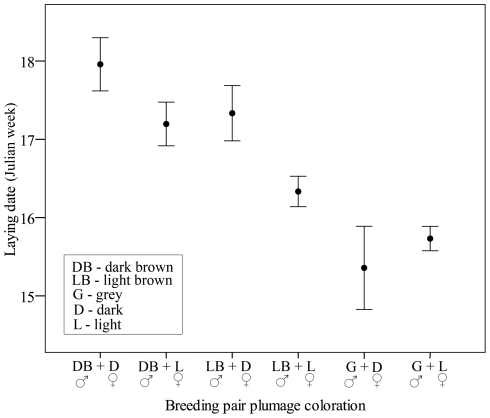
Mean laying date of breeding pairs of marsh harriers composed of individuals with different plumage colorations (Ebro basin, NE Spain, 2001–2008).

From 2001 to 2008 we monitored 290 breeding attempts of marsh harriers, with complete data on productivity and male and female coloration available for 219 of them (76% of the cases). Annual breeding population in those years was 36±9 breeding pairs (mean ± standard deviation, range 25–53). Each year the breeding population was composed of 15% (±12%) of dark brown males, 32% (±17%) of light brown males and 53% (±13%) of grey males. Breeding females included 13% (±5%) of dark and 87% (±4%) of light birds (mean ± SD, n = 8 years). No significant differences among plumage color scores assigned by different observers to the same birds were found (F_4,75_ = 0.08, P = 0.99), meaning that we can be confident about accuracy of color assessments. Sexes did not mate randomly in relation to plumage coloration (Spearman's rank correlation coefficient, r_s_ = 0.3, P<0.001), mating between dark individuals (dark brown males and dark females) or light individuals (grey males and light females) being more frequent than crossed mating (Fig. 2). Laying date strongly varied among breeding pairs, those formed by grey males and light females starting earlier than pairs composed of two dark birds (dark brown males and dark females, Fig. 3).

### Habitat heterogeneity, bird coloration and breeding performance

Productivity of breeding pairs was not significantly different among localities (F_41,169_ = 0.95, P = 0.5667), but varied according to the plumage coloration of their members. When considering both members of the pair simultaneously in models, the main effect of male coloration was positively linked to productivity (F_1,208_ = 9.40, P = 0.0025; i.e., pairs formed by brown males were less successful than those composed by lighter males, mean productivity ± SD: dark brown males, 0.82±1.24; light brown males, 1.03±1.28; grey males, 1.50±1.28) while the main effect of female coloration on productivity was non-significant (F_1,208_ = 1.06, P = 0.3049; mean productivity ± SD: dark females, 0.86±1.19; light females, 1.32±1.31). However, the interaction between plumage coloration of both members was significant (F_1,166_ = 3.79, P = 0.0532), supporting that breeding pairs formed by grey males and light females raised significantly more fledglings than pairs including at least one individual of the other coloration categories or pairs formed by two dark birds. All of these relationships arose while controlling for polygyny (range of P-values for different models: 0.11–0.37) and year as a random term (range of covariance parameter estimates for different models: 8.47*10^−20^–1.07*10^−18^).

### Bird coloration and conspecific density

**Figure 4:**
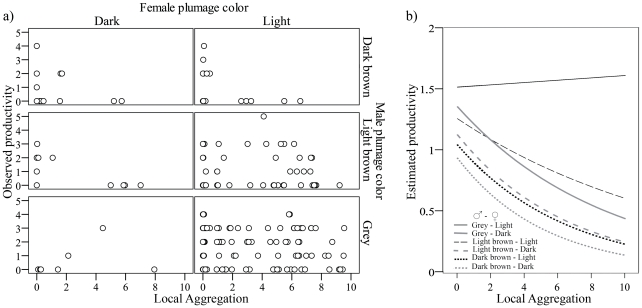
Interactive effects of conspecific aggregation and plumage coloration of breeding males and females on productivity. (a) Raw data and (b) relationship derived from fitted values from GLMM estimates (poisson error distribution, log link function) are shown.

Neither the single linear effect of local aggregation of conspecifics nor its quadratic form were significant in models (all P values>0.42), apparently not supporting the existence of density-dependent effects on productivity. However, there was a significant interaction between male and female plumage coloration and local aggregation (*local aggregation×female×male*: F_1,165_ = 5.63, P = 0.0188) suggesting that the relationship between productivity and density of conspecifics was complex and linked to breeder coloration, with no apparent effect of habitat characteristics (i.e. locality, F_41,165_ = 089, P = 0.67). In the study population, productivity of brown males (both dark and light) decreased at high breeding densities, an effect that was more marked when these birds were paired with dark females. Contrarily, productivity of grey males and light females remained unchanged along the local breeding density gradient (Fig. 4a,b). This relationship arose while controlling for polygyny as a fixed effect (P = 0.18) and year as a random term (covariance parameter estimate: 1.59*10^−19^) in the model.

### Bird coloration, settlement pattern and population expansion

**Figure 5:**
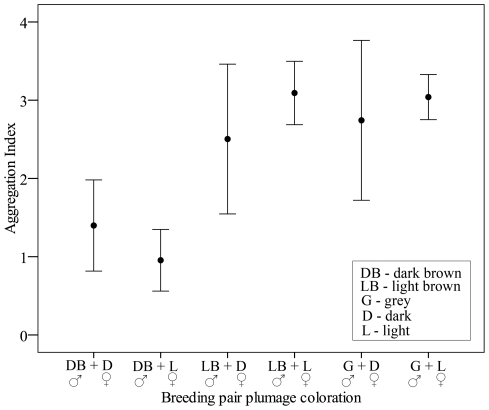
Relationship between local aggregation index and breeding pair plumage coloration of the marsh harrier (Ebro basin, NE Spain, 2001–2008).

Local conspecific aggregation (i.e., aggregation of breeding marsh harriers within breeding sites) varied among breeding pairs with different plumage coloration, when controlling for polygyny (p<0.001) as a fixed effect and year as a random term (covariance parameter estimate: 0.08) in models. Breeding pairs including dark brown males nested more isolated than pairs including lighter males (F_1,199_ = 4.89, p = 0.028) (Fig. 5). The effect of female coloration, which was included simultaneously in the model, was non-significant (F_1,199_ = 0.01, P = 0.92). The interaction between male and female plumage coloration was neither significant (F_1,198_ = 0.02, p = 0.88).

**Figure 6:**
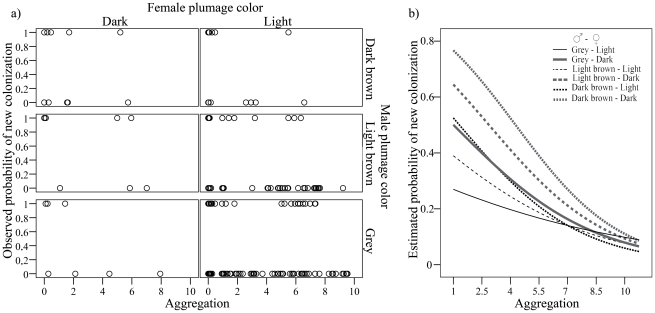
Relationship between population aggregation and probability of occupancy of new nesting sites by breeding pairs of marsh harriers with different colorations. (a) Raw data and (b) relationship derived from fitted values from GLMM estimates (binomial error distribution, logit link function) are shown.

When modeling the probability of occupation of new localities, we found a significant interaction between male and female coloration and population aggregation (F_1,165_ = 4.93, P = 0.0278), suggesting that the probability of colonization of empty sites could be related to breeder coloration and to their relative position within the breeding population (i.e., population aggregation), with no apparent effect of characteristics of habitat patches (‘locality’, F_41,165_ = 0.52, P = 0.99). Thus, the colonization of the most isolated, newest nesting sites seems to be mainly associated with settlement of breeding pairs formed by dark birds (Fig. 6a, b). This relationship arose while controlling for the non-significant effects of polygyny (P = 0.10). Year was included as a random term (covariance estimate: 13.67).

## Discussion

Spatially-structured social aspects such as the presence and the number of conspecifics are important sources of spatial heterogeneity that might affect both breeding performance and occupancy patterns on birds [5], [21], [44]–[46]. In our study, productivity of breeding marsh harriers varied in relation to local breeding densities, with strong differences among individuals with different plumage coloration (i.e., phenotypes) and no apparent effects of habitat patches. In this way, local breeding densities combined with individual variability seem to be an important determinant of breeding output in this species, suggesting that not all individuals had the same expectation of success at a given population density. In a similar way, our results showed an uneven occupation of localities by individuals with different plumage coloration in relation to local densities, lighter harriers breeding in a more aggregated fashion than dark-brown ones. The segregation of birds with different traits in patches of high or low conspecific densities is probably the result of individuals sorting themselves out to maximize their own expected fitness [16].

The final effect of conspecific density on productivity might be the result of a balance between density-dependent costs and benefits. Individual traits might thus influence individual net gain at different population densities while optimal aggregation might be different for individuals with different traits [16], [21]. In our study, the decrease in breeding output of brown breeders as population aggregation increased suggests that in high density situations their low level of aggressiveness (resulting from differences in melanism or age) [19], [26], [47] may decrease their competitive abilities when they interact with neighbors [21], [48], thus reducing their breeding performance. In fact, conspecific presence is associated with increased costs of mate guarding and paternity assurance in other harrier species [49], [50], and possibly increased competition for food [12]. Contrarily, more competitive lighter birds might benefit by settling near conspecifics since groups are more effective at territory defence or food searching [5], [11], [12]. The main expected benefit of large group size can be linked to the other, unexplored, fitness parameter, i.e. individual survival, with isolated breeding pairs of colonial species being more prone to predation than those breeding in colonies, as well as with increased mating opportunities and facilitation of mate choice [5], [11], [46].

Our results thus suggest that the relationship between fecundity and density is neither linear or monotonic [7] but variable among individuals depending on their characteristics [15], [20]. Indeed, our study model did not show a relationship between the single effect of aggregation and productivity but resulted in a significant effect of its interaction with bird coloration. This result highlights that some traditional studies which only consider the overall population, without taking into account differences among individuals [7], [45], could have overlooked the role of density-dependence on population regulation as well as on settlement patterns [15].

Positive density-dependence, acting on a part of the population (i.e. lighter harriers), could explain the existence and maintenance of areas supporting high population densities, even if they were not of consistently different quality than surrounding areas [15], [16]. Regarding the scenario of population expansion, intraspecific competition acting on another part of the population (i.e. darker harriers) could also promote that empty isolated patches became occupied by this part of the population. For less competitive birds, the benefits of settling in traditional areas may be counterbalanced by the costs of intraspecific competition, making dispersal to unoccupied areas advantageous [15]. This mechanism could be promoting the increment in the population range and, therefore, its expansion.

When studying population expansions, phenotypic plasticity, i.e. the capacity of a given genotype to produce different phenotypes under different environmental conditions, has been the focus of many studies [51]–[53]. For example, behavioral flexibility has been proposed as one of the main mechanisms to explain the invasion of novel habitats by invasive species because it provides them with the ability to expand or change their ecological niche [54]–[56]. Recently, however, the role of phenotypic variability in populations during range expansions has been reinterpreted more as a result of variability among individuals, rather than within individuals [57]. Thus, the traits of individuals, but not the average trait at the level of species, are important during the occupation of new habitats [58], [59].

Melanin pigments (grey-black eumelanin-based versus rufus-brown pheomelanin based plumages) provide a widespread source of coloration in vertebrates and it has been argued that their expression is mainly controlled by genes [22]–[24]. Yet, the adaptive significance of such genetic color polymorphism remains poorly understood. However, the findings that melanin-based colors frequently co-vary with life-history traits [22]–[24] and that genes coding for the expression of melanin pigmentation have pleiotropic effects on the expression of other physiological processes, such as immune functions or oxidative stress [23], has led to the hypothesis that differently colored individuals are adapted to alternative environments varying in food abundance, social interactions or parasite exposure [22], [60]. Thinking of the expansion of the marsh harrier, a similar situation can be portrayed, with individuals of different melanin-based colorations following alternative strategies [26]. Brown individuals avoiding high-density situations colonize new, little populated patches, promoting the expansion of the species, while grey ones distribute more frequently in previously-used, highly populated ones. Fogarty et al. [61] proposed that within-species variation in behavioural traits and behavioural dependent dispersal can have important effects on invasion dynamics. Polymorphism in sociability and aggressiveness (which are usually personality types) increases the speed of the invasion front, since asocial or less aggressive individuals colonize empty patches and facilitate the local growth of social or aggressive types that, in turn, induce faster dispersal by asocial or less aggressive individuals at the invasion edge. Although not focused on individual personalities, our results are highly in accordance with predictions of the theoretical model of Fogarty et al. [61], offering preliminary empirical support and highlighting the importance of continuing this interesting line of research.

Since in the marsh harrier older birds also tend to be lighter, an alternative explanation to the pattern found in this study could be related with variation in competitive skills with age [47], [48]. Adult birds (i.e., light birds) can counterbalance the negative effects of crowding by taking advantage of communal defense [12], while younger individuals (i.e., dark birds) suffer more the cost of overcrowding than the benefits of communal defense. Expanding populations are commonly characterized by a high proportion of non-adult breeders [40], [62], a phenomenon explained by the presence of vacant habitat patches available for nesting (but not necessarily of high habitat quality) where these low competitive individuals can breed. This could be occurring in our study area, where agricultural intensification in last decades has lead to an increase in artificial irrigation ponds and reservoirs surrounded by herbaceous crops, with available food, suitable for the nesting of the species [31], [63]. Also in this case, variability in animal traits might contribute to variation in age-specific fecundity across individuals, if reproduction at an early age is not only the result of ecological factors acting on individuals, but is also the result of intrinsic and genetic differences between individuals [64]. In this situation, the higher reproductive output expected for young breeders taking advantage of little populated localities can favor the selection of individuals breeding at younger ages [64], thus increasing the proportion of the population looking for such isolated habitat patches. Although more research is needed to fully understand expansions, evolutionary processes at range limits combined with density-dependent dispersal should be considered as important drivers of distribution changes in this and other species [61], [65], [66].
